# Corrigendum to “A systematic review of the impact of compression therapy on quality of life and pain among people with a venous leg ulcer”

**DOI:** 10.1111/iwj.14892

**Published:** 2024-05-03

Patton D, Avsar P, Sayeh A, et al. A systematic review of the impact of compression therapy on quality of life and pain among people with a venous leg ulcer. *Int Wound J*. 2024;21(3):e14816. doi: 10.1111/iwj.14816.

Rosemarie Derwin was omitted from the list of authors in error. Here is the amended list of authors:

[Declan Patton^1,2,3,4,5^ | Pinar Avsar ^1^ | Aicha Sayeh^1^ | Aglecia Budri ^2^ | Tom O'Connor ^1,2,3,4,6^ | Simone Walsh^7^ | Linda Nugent ^2,3^ | Denis Harkin^8^ | Niall O'Brien^9^ | Jonathan Cayce^10^ | Michael Corcoran^10^ | Mario Gaztambide^10^ |Rosemarie Derwin^1,2^| Zena Moore^1,2,3,4,11,12,13,14^].

We apologize for this error.

